# Annotated checklist of Scoliidae (Hymenoptera, Scolioidea) of the Americas

**DOI:** 10.3897/zookeys.1285.198549

**Published:** 2026-07-22

**Authors:** Luis Damián Ramírez-Guillén, Jorge L. León-Cortés, Luc Leblanc, Armando Falcon-Brindis

**Affiliations:** 1 Posgrado en Ciencias en Ecología y Desarrollo Sustentable, El Colegio de la Frontera Sur, Carretera Panamericana y Periférico Sur S/N C.P. 29290, San Cristóbal de las Casas, Chiapas, Mexico Department of Entomology, Plant Pathology and Nematology, William F. Barr Entomological Museum, University of Idaho Moscow United States of America; 2 Departamento de Conservación de la Biodiversidad, El Colegio de la Frontera Sur, Carretera Panamericana y Periférico Sur S/N C.P. 29290, San Cristóbal de las Casas, Chiapas, Mexico Posgrado en Ciencias en Ecología y Desarrollo Sustentable, El Colegio de la Frontera Sur San Cristóbal de las Casas Mexico; 3 Department of Entomology, Plant Pathology and Nematology, William F. Barr Entomological Museum, University of Idaho, 875 Perimeter Drive, MS 2329, Moscow, Idaho 83844-2329, USA Departamento de Conservación de la Biodiversidad, El Colegio de la Frontera Sur San Cristóbal de las Casas Mexico; 4 Department of Entomology, Plant Pathology and Nematology, Parma Research and Extension Center, University of Idaho, 29603 U of I Lane, Parma, Idaho 83660, USA Department of Entomology, Plant Pathology and Nematology, Parma Research and Extension Center, University of Idaho Parma United States of America

**Keywords:** Campsomerini, Scoliini, species diversity, taxonomy, Trielidina

## Abstract

A total of 72 species in 14 genera of Scoliidae are listed for the Americas. The countries with the highest number of species were Brazil (*n* = 32), Mexico (29), and the United States (24), while the country with the lowest number was Saint Lucia (1). The genus *Triscolia* are restricted to North America. *Sphenocampsomeris* and *Tenebromeris* are restricted to South America. *Aelocampsomeris variegata*, *Campsomeris peregrina*, *Dielis dorsata*, *Pygodasis ephhipium*, *Stygocampsomeris servillei* have the widest distribution, from Mexico to South America. An updated taxonomic classification of Scoliidae species in the Americas is presented, providing an important baseline for future taxonomic and conservation studies. Further research should prioritize the scoliid fauna of underrepresented countries in Central and South America.

## Introduction

The family Scoliidae (Hymenoptera: Scolioidea) comprises approximately 560 species in 43 genera worldwide (Osten 2005). Scoliidae is divided into three subfamilies: Archaeoscoliinae, Scoliinae and Proscoliinae. Archaeoscoliinae is an extinct subfamily comprising ten known fossil species (Nel et al. 2013). Within Proscoliinae, two species have been described, both from the Palearctic region (Day et al. 1981). Scoliinae is divided into two tribes, Campsomerini and Scoliini, both of which are present in the Americas. The classification of Scoliidae has been regarded as poorly developed, with many ambiguous descriptions (Day et al. 1981; Argaman 1996). This situation is similar in the Americas, where the first significant contribution was made by Bartlett (1912), focusing on Scoliini. Subsequently, Bradley and Betrem produced numerous publications addressing Campsomerini and revising several genera of Scoliidae (Bradley 1927, 1928a, 1928b, 1940, 1945, 1957, 1964a, 1964b, 1973, 1974; Betrem 1962, 1963; Bradley and Betrem 1966, 1967). Despite such efforts, these works lacked comprehensive descriptions of species and focused on South and Central America. In North America, specifically in the United States, the scoliid fauna has been addressed in multiple regions, but with similar issues, i.e., vague keys and species descriptions (Hurd 1952; Porter 1981; MacKay 1987; Finnamore and Hanson 1995; s). In recent years, the study of Scoliidae in the Americas has increased, resulting in numerous taxonomic revisions, new distributional records, and species descriptions (Añino et al. 2020, 2024; Khouri et al. 2022; Ramírez-Guillén and Falcon-Brindis 2022; Ramírez-Guillén et al. 2022, 2025a, 2025b, 2025c, 2026; Golfetti and Noll 2023a, 2023b; Szafranski 2023, 2026; Castagnet et al. 2025; Golfetti et al. 2025).

Given the growing attention to these wasps and the complex taxonomic history of Scoliidae, it is necessary to compile and update the literature on American Scoliidae to prevent future taxonomic confusion. Here, we present an updated species list of Scoliidae across countries in the Americas and highlight current knowledge gaps. We also correct taxonomic inconsistencies identified in previous local and regional surveys, thereby establishing a reliable baseline of genera and species in the Americas.

## Materials and methods

To establish the new checklist with valid names, we followed the most up-to-date classifications provided by Osten (2005) to species and Taylor (2024) to genera, subtribes, and tribes. The species with doubtful scientific names (based on Osten 2005) are listed but not included in the total number of species for the Americas. For each species, information about the distribution is provided, along with synonyms when applicable. The first territorial division (i.e., state or province) is provided, then the subsequent division is placed in parentheses when the record or label did not mention the first territorial division. An asterisk “*” is added to identify new records, and a question mark “?” when the provided information is doubtful.

From 2024 and 2026, a total of 7,325 specimens were examined from the following collections:

**WFBM** University of Idaho, William F. Barr Entomological Museum, Moscow, ID;

**CIDA** College of Idaho, Orma Smith Museum of Natural History, Caldwell, ID;

**FSCA** Florida State Collection of Arthropods, Gainesville, FL;

**OSDA** Oregon State Department of Agriculture Collection, Wilsonville, OR;

**OSAC** Oregon State Collection of Arthropods, Corvallis, OR;

**WSUC** James Entomological Museum, Washington State University, Pullman, WA;

**ICUAP** Benemérita Universidad Autónoma de Puebla, Instituto de Ciencias, Colección Entomológica Miguel Ángel Morón Ríos;

**UATx** Universidad Autónoma de Tlaxcala, Colección Biológica;

**CZUG** Universidad de Guadalajara, Colección entomológica del Centro de Estudios en Zoología;

**IEXA** Instituto de Ecología A.C., Colección Entomológica;

**CIBE** Universidad Autónoma de Nuevo León, Facultad de Ciencias Biológicas, Colección de Insectos Benéficos y Entomófagos;

**CAFESI** Universidad Nacional Autónoma de México, Facultad de Estudios Superiores Iztacala, Colección de Artrópodos;

**CNIN** Universidad Nacional Autónoma de México, Instituto de Biología, Colección Nacional de Insectos;

**UVGC** Universidad del Valle de Guatemala, Centro de Estudios Ambientales y Biodiversidad, Colección de Artrópodos;

**CANG** Universidad de San Carlos de Guatemala, Centro de Estudios Conservacionistas, Colección de Abejas Nativas de Guatemala;

**EAPZ** Escuela Agrícola Panamericana, Universidad Zamorano, Colección de Insectos de Zamorano;

**MNCR** Colección de artrópodos del Museo Nacional de Costa Rica;

**MZCR** Universidad de Costa Rica, Escuela de Biología, Museo de Zoología;

**MUCR** Universidad de Costa Rica, Escuela de Agronomía, Museo de Insectos.

The geographical region is also provided for each species using the abbreviated form: Antilles (**ANT**), Central America (**CAM**), North America (**NAM**), and South America (**SAM**). For the abbreviation of countries, we used ISO 3166-1 alpha-3.

## Results

A total of 72 species in 14 genera of Scoliidae were listed. There were five species bearing doubtful names (nomen dubium). For the Tribe Campsomerini, 56 species in 12 genera were found, and 16 species in two genera for the Tribe Scoliini. The genera *Colpa* and *Crioscolia* are restricted to North America, and *Scolia* is distributed across the Americas. The remaining 11 genera are endemic to the Americas. *Triscolia* are restricted to North America. *Sphenocampsomeris* and *Tenebromeris* are restricted to South America. *Pygodasis* and *Rhabdotomeris* are not found in the Antilles. *Aelocampsomeris*, *Campsomeris*, *Dielis*, *Lissocampsomeris*, *Stygocampsomeris* and *Xanthocampsomeris* are distributed across the Americas (Table 1).

**Table 1.** Proportion and species richness of scoliids across geographic regions of the Americas. Number of species (S). North America (NAM), Central America (CAM), South America (SAM), Antilles (ANT). Taylor’s 2024 classification of genera, subtribes, and tribes.

**Tribe**	**Subtribe**	**Genus**	**S**	**%**	**Geographic region**
Campsomerini	Campsomerina	* Aelocampsomeris *	3	4	NAM, CAM, ANT, SAM
		* Campsomeris *	4	5	NAM, CAM, ANT, SAM
		* Dielis *	12	17	NAM, CAM, ANT, SAM
		* Lissocampsomeris *	7	10	NAM, CAM, ANT, SAM
		* Pygodasis *	14	19	NAM, CAM, SAM
		* Rhabdotomeris *	1	1	NAM, CAM, SAM
		* Sphenocampsomeris *	1	1	SAM
		* Stygocampsomeris *	4	6	NAM, CAM, ANT, SAM
		* Tenebromeris *	1	1	SAM
		* Xanthocampsomeris *	5	7	NAM, CAM, ANT, SAM
	Trielidina	* Colpa *	2	3	NAM
		* Crioscolia *	2	3	NAM
Scoliini		* Scolia *	14	19	NAM, CAM, ANT, SAM
		* Triscolia *	2	3	NAM
Total		14	72	99	

**Figure 1.**
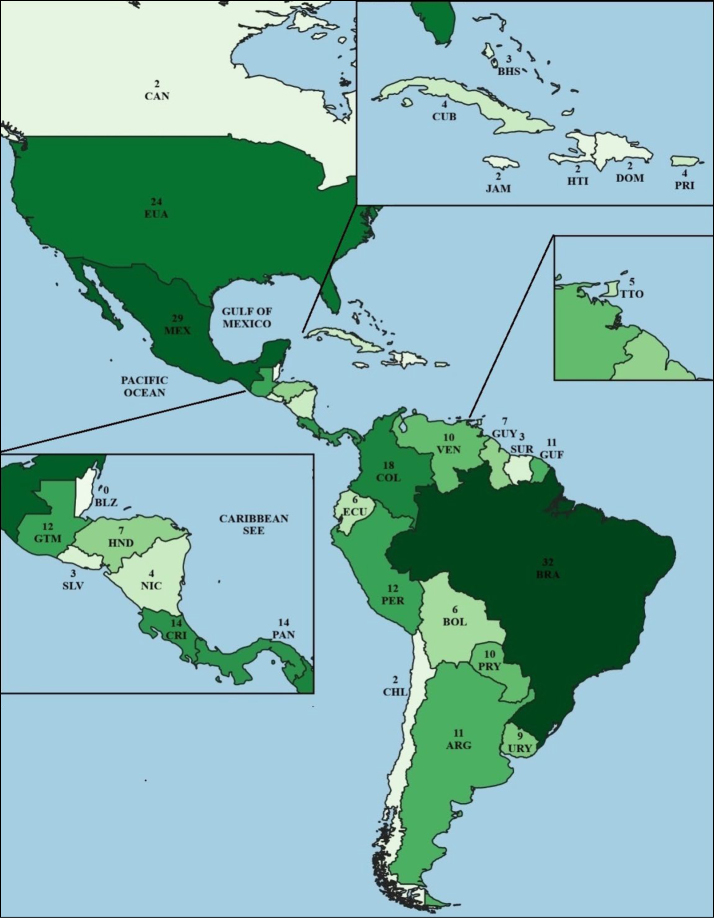
Species of Scoliidae across the Americas. Names of countries in ISO 3166-1 alpha-3.

The highest number of species was recorded in Brazil (*n* = 32), Mexico (29), and the United States (24) (Fig. 1). The countries with the lowest number of records were Canada, Chile, Dominican Republic, Jamaica, and Saint Kitts and Nevis, each with two species, and Saint Lucia with a single species. Belize was the only country with no records yet. The species with the widest distribution in the Americas was *Pygodasis ephhipium*, extending from the southern United States (Texas) to Argentina, *Aelocampsomeris variegata* from northern Mexico (Coahuila) to southern Brazil (Rio Grande do Sul), *Campsomeris peregrina* from Mexico to southern Argentina (Buenos Aires), *Stygocampsomeris servillei* from southern Mexico (Chiapas) to Uruguay, and *Dielis dorsata* from southern Mexico (Chiapas) to Paraguay (introduced in Florida, United States). Endemism has been reported in Brazil (*n* = 6), the United States (3), Mexico (2), Argentina (2), the Bahamas (1), French Guiana (1), Peru (1), and Uruguay (1) (Table 2).

**Table 2.** Endemic species by country. Number of species (*n*).

**Country**	***n***	**Species**
Brazil	6	*Dielis auripilis*, *Dielis diabo*, *Dielis pseudonyma*, *Stygocampsomeris manauara*, *Tenebromeris tenebrica*, and *Scolia binominata*
United States	3	*Dielis tejensis*, *Pygodasis quadrimaculata*, and *Scolia bicincta*
Mexico	2	*Scolia bartletti* and *Scolia fuscipennis*
Argentina	2	*Pygodasis spegazzini* and *Scolia scutata*
Panama	2	*Lissocampsomeris bribri* and *Scolia albumtenebris*
Bahamas	1	*Dielis bahamensis*
French Guiana	1	*Lissocampsomeris idonea*
Peru	1	*Dielis whitelyi*
Uruguay	1	*Pygodasis cineraria*

### Family: Scoliidae Latreille, 1802

**Subfamily: Scoliinae Latreille, 1802**

**Tribe: Campsomerini Bartlett, 1912**

**Subtribe: Campsomerina Bartlett, 1912**

**Genus: *Aelocampsomeris* Bradley, 1957**

#### *Aelocampsomeris brethesi* Bradley, 1945

**Distribution**. ANT, CAM, SAM. Bolivia (eastern Andes). Brazil: São Paulo. Colombia: Meta. Costa Rica*: Alajuela, Guanacaste, Heredia, San José. French Guiana. Guyana. Panama. Peru: Arequipa* Loreto. Trinidad and Tobago (Trinidad Island) (Bradley 1927, 1945; Fernández and Cubillos 1999).

#### *Aelocampsomeris campestris* Burmeister, 1854

**Distribution**. SAM. Argentina. Brazil: Minas Gerais (Schrottky 1910; Bradley and Betrem 1966).

**Synonym**. *Elis pulchella* Saussure, 1855.

#### *Aelocampsomeris variegata* Fabricius, 1793

**Distribution**. ANT, CAM, NAM, SAM. Argentina: Misiones. Bolivia (east of the Andes). Brazil: Bahía, Mato Grosso do Sul (Chapada), Rio de Janeiro, Rio Grande do Sul, São Paulo. Colombia: Meta, Santander, Tolima. Costa Rica: Alajuela*, Heredia*, Guanacaste*, Puntarenas*, San José*. Mexico: Coahuila, Querétaro, Veracruz (Orizaba). Guatemala. French Guiana: Cayennne. Guyana. Panama: Coclé*, Panamá, Panamá Oeste*. Paraguay: Alto Paraná (Puerto Bertoni). Peru: Arequipa*, Junín, Madre de Dios*. Surinam. Trinidad and Tobago. Venezuela: Aragua, Distrito Capital (Caracas) (Cameron 1893; Schrottky 1910; Bradley 1940, 1945; Finnamore and Hanson 1995; Fernández and Cubillos 1999; Dos Santos et al. 2015; Ramírez-Guillén et al. 2022).

**Synonyms**. *Scolia fuscata* Klug, 1810; *Campsomeris lucida* Lepeletier, 1845; *Camposmeris costalis* Lepeletier, 1845; *Scolia irregularis* Smith, 1855; *Elis lativentris* Saussure, 1855.

### Genus: *Campsomeris* Guérin-Méneville, 1838

#### *Campsomeris atrata* Fabricius, 1775

**Distribution**. ANT. Bahamas: North Eleuthera*. Cuba: Holguín*, Santiago de Cuba. Dominican Republic*: La Vega, Pedernales. North Eleuthera*. Puerto Rico: Aguadilla/Mayagüez, Guayama (Aguirre), Ponce (Gundlach 1887; Wolcott 1948; Fernández et al. 2001; Portuondo-Ferrer and Fernández-Triana 2004).

#### *Campsomeris peregrina* Lepeletier, 1845

**Distribution**. CAM, NAM, SAM. Argentina: Buenos Aires: Chaco, Misiones. Bolivia. Brazil: Mato Grosso do Sul (Chapada). Colombia: Bogotá, Cundinamarca, Meta, Tolima Valle del Cauca. Costa Rica*: Limón, Puntarenas. Ecuador. Guyana. Honduras. Mexico: Guerrero*, Veracruz*. Paraguay. Peru. Venezuela: Distrito Capital (Cameron 1893; Fox 1896; Schrottky 1910; Bradley 1940, 1945, 1964b, 1974; Fernández and Cubillos 1999).

**Synonyms**. *Elis regina* Saussure, 1858; *Elis regalis* Sichel, 1864.

#### *Campsomeris regifica* Bradley, 1945

**Distribution**. CAM, SAM. Brazil: Amazonas. Colombia: Cundinamarca. Costa Rica: Alajuela*, Cartago*, Guanacaste*, Heredia*, Limón*, San José*. French Guiana: Saint-Laurent-du-Maroni. Guyana: Cuyuní-Mazaruní (Kartabo). Guatemala*: Guatemala. Peru: Huánuco, Loreto (Bradley 1945; Finnamore and Hanson 1995; Fernández and Cubillos 1999; Rasmussen and Asenjo 2009).

#### *Campsomeris vitripennis* Smith, 1855

**Distribution**. CAM, NAM, SAM. Argentina: Chaco, Córdoba, Jujuy, La Rioja, Mendoza, Santa Fe, Santiago del Estero, Tucumán. Brazil: Amazonas, Mato Grosso. French Guiana: Cayenne. Honduras*: Francisco Morazán. Mexico: Guerrero, Veracruz*. Peru*: Madre de Dios (Cameron 1893; Fox 1896; Schrottky 1910; Bradley 1940, 1964a, 1964b; Bradley and Betrem 1967).

**Synonyms**. *Colpa infuscata* Lepeletier, 1845; *Scolia subobscura* Smith, 1845; *Campsomeris lucida* Bradley, 1927; *Campsomeris luciflua* Bradley, 1940.

### Genus: *Dielis* Saussure & Sichel, 1864

#### *Dielis auripilis* Fox, 1896

**Distribution**. SAM. Brazil: Mato Grosso do Sul (Chapada) (Fox 1896).

#### *Dielis bahamensis* Bradley, 1964

**Distribution**. ANT. Bahamas: Acklins, Island of Crooked, Inagua, Island of Larga (Bradley 1964a).

#### *Dielis chilensis* Saussure, 1858

**Distribution**. SAM. Chile. Peru: Cusco (Bradley 1974; Rasmussen and Asenjo 2009).

#### *Dielis diabo* Golfetti & Noll, 2023

**Distribution**. SAM. Brazil: São Paulo (Golfetti and Noll 2023a).

#### *Dielis dorsata* Fabricius, 1787

**Distribution**. ANT, CAM, NAM, SAM. Bolivia*: Beni, La Paz, Santa Cruz. Brazil: Amazonas*, Mato Grosso, Mato Grosso do Sul (Chapada, Corumba), Pará (Santarem), Rio de Janeiro, Santa Catarina*, São Paulo. Dominican Republic: Santo Domingo. Colombia: Antioquia, Arauca, Bolívar, Casanare, Cauca*, Meta, Nariño, Tolima, Valle del Cauca, Vichada. Costa Rica: Puntarenas*. Ecuador*: Imbabura. El Salvador: La Libertad*, San Salvador. Guatemala: Huehuetenango. Mexico: Chiapas, Colima, Quintana Roo, Veracruz. Nicaragua: Rivas. Panama: Bocas del Toro*, Coclé*, Colón*, Panamá, Panamá Oeste*. Paraguay: Alto Paraná (Puerto Bertoni, Tacurú Pucú), Itapúa (Encarnación). Peru: Loreto. Puerto Rico: Arecibo (Dorado), Carolina (Trujillo), Ponce (Guanica), San Juan (Río Piedras). Saint Kitts and Nevis: (Saint Kitts Island). United States: Florida (Introduced in United States). Venezuela: Distrito Capital (Antimano, Caracas, El valle), La Guaira (Galipán). West Indies to Paraguay (Gundlach 1887; Fox 1896; Schrottky 1910; Bradley 1928b, 1945; Wolcott 1948; Finnamore and Hanson 1995; Fernández and Cubillos 1999; Rasmussen and Asenjo 2009; Abbate et al. 2018; Aranda and Ie 2019; Ramírez-Guillén et al. 2022).

**Synonyms**. *Scolia haematogastra* Perty, 1833; *Colpa rubida* Lepeletier, 1845; *Elis sabulosa* Saussure, 1858; *Scolia reversa* Schulz, 1906; *Elis pygmaea* Schrottky, 1910.

#### *Dielis pilipes* Saussure, 1858

**Distribution**. NAM. Canada: British Columbia. Mexico: Tamaulipas. United States: Arizona, California, Colorado, Idaho, Kansas, Nebraska*, Nevada, New Mexico, Oregon, South Dakota, Texas, Utah, Washington, Wyoming (Bradley 1928b; Hurd 1952; Ruíz et al. 2010; Nelson and Moffat 2021).

**Note**. This taxon has been in dispute. Bradley (1957) divided the genus *Dielis* into three groups (*pilipes*, *plumipes* and *psuedonyma*). Only *D. pilipes* is grouped in *pilipes*. Betrem (in Bradley 1964a) suggested that this species should form a separate group. Khouri et al. (2022), proposed that *D. pilipes* is closely related to *X. limosa*. He did not review all the taxa of *Xanthocampsomeris* and to date, this work remains in press.

#### *Dielis plumipes plumipes* Drury, 1770

**Distribution**. NAM. United States: Connecticut, Florida*, Georgia, Kentucky, Maryland, Massachusetts, Missouri*, New Jersey, New York, North Carolina, Pennsylvania, Rhode Island, Tennessee*, Texas*, Virginia (Bradley 1928b).

**Synonyms**. *Scolia quadricincta* Klug, 1805; *Scolia radula* Fabricius, 1775.

#### *Dielis plumipes confluenta* Say, 1823

**Distribution**. NAM. Canada: Ontario. United States: Arkansas, Colorado, Illinois, Indiana, Kansas, Michigan, Minnesota, Nebraska, New Mexico, Ohio, South Dakota, Wisconsin (Bradley 1928b).

#### *Dielis plumipes fossulana* Fabricius, 1805

**Distribution**. NAM. Mexico: Tamaulipas?. United States: Alabama, Arkansas, Florida, Georgia, Kansas, Louisiana, Mississippi, North Carolina, South Carolina, Texas (Bradley 1928b; Grissell 2007; Ruíz et al. 2010; Szafranski 2026).

#### *Dielis pseudonyma* Schulz, 1906

**Distribution**. SAM. Brazil: Mato Grosso do Sul (Corumbá) (Fox 1896).

**Synonym**. *Elis smithii* Fox, 1896.

#### *Dielis tejensis* Szafranski, 2023

**Distribution**. NAM. United States: Texas (Szafranski 2023, 2026).

#### *Dielis tolteca* Saussure, 1857

**Distribution**. ANT, CAM, NAM. El Salvador: San Salvador. Guatemala: Huehuetenango. Honduras: Comayagua. Haiti: west (Port-au-Prince). Mexico: Baja California, Baja California Sur, Chihuahua, Coahuila, Guanajuato, Guerrero, Jalisco, Michoacán, Morelos, Oaxaca, Quintana Roo, Sinaloa, Sonora, Tabasco, Tamaulipas, Veracruz, Yucatán. Nicaragua: Rivas. Panama. United States: Arizona, California, New Mexico, Texas, Utah* (Saussure 1857; Bradley 1928b; Hurd 1952; Porter 1981; MacKay 1987; Ramírez-Guillén et al. 2022; Añino et al. 2024).

**Synonyms**. *Elis dives* Provancher, 1888; *Elis quadricincta* Provancher, 1888.

#### *Dielis trifasciata trifasciata* Fabricius, 1793

**Distribution**. ANT, NAM. Cuba: Cienfuegos (Soledad), Guantánamo, Holguín (Preston), La Habana, Pinar del Río*, Santiago de Cuba. Haiti: Oeste (Bizoton, Fond Parisien Port-au-Prince), Sur (Aux Cayes, La Moriniere). Jamaica: Manchester (Mandeville), Portland, Saint Andrew (Linguanea Plain). Puerto Rico: Aguadilla/Mayagüez, Arecibo (Manatí), Bayamón, Guayama (Aguirre) Humacao (Yabucoa), San Juan (Rio Piedras). Saint Kitts and Nevis: (Saint Kitts Island). Saint Lucia. United States: Florida (Gundlach 1887; Bradley 1928b; Wolcott 1948; Fernández et al. 2001; Portuondo-Ferrer and Fernández-Triana 2004; Grissell 2007).

**Synonyms**. *Colpa alexandri* Lepeletier, 1845; *Elis pygmaea* Schrottky, 1910?; Genus? *reversa* Schulz, 1906?.

#### *Dielis trifasciata nassauensis* Bradley, 1928

**Distribution**. ANT. Bahamas: Cat Island (on the island of Little San Salvador), New Providence (Nassau), North Eleuthera*, San Salvador Island* (Bradley 1928b).

#### *Dielis whitelyi* Kirby, 1889

**Distribution**. SAM. Peru: Arequipa (Valle de Tambo), Cusco (Kirby 1889; Rasmussen and Asenjo 2009).

### Genus: *Lissocampsomeris* Bradley, 1957

#### *Lissocampsomeris argentea* Haliday, 1837

**Distribution**. SAM. Brazil: Rio Grande do Sul, São Paulo. Uruguay: Lavalleja (Minas), Montevideo (Haliday 1837; Saussure 1858; Dalla Torre 1897; Bradley 1974).

**Synonyms**. *Elis brasiliana* Saussure, 1858; *Elis gerstaeckeri* Saussure, 1858.

#### *Lissocampsomeris aureohirta aureohirta* Fox, 1896

**Distribution**. SAM. Brazil: Mato Grosso do Sul (Chapada) (Fox 1896).

#### *Lissocampsomeris aureohirta ecuadorensis* Cameron, 1893

**Distribution**. SAM. Peru: Junín, Ucayali (Dos Santos et al. 2015).

#### *Lissocampsomeris bribri* Ramírez-Guillén, 2026

**Distribution**. CAM. Panama: Panama (Ramírez-Guillén et al. 2026).

#### *Lissocampsomeris columba columba* Saussure, 1858

**Distribution**. CAM, NAM, SAM. Colombia: Valle del Cauca. Guatemala: Alta Verapaz, Baja Verapaz, Izabal. Mexico: Chiapas and Veracruz. Venezuela: Distrito Capital (Caracas) (Saussure 1858; Dalla Torre 1897; Bradley 1945; Fernández and Cubillos 1999; Ramírez-Guillén et al. 2026).

#### *Lissocampsomeris columba albofimbriata* Smith, 1879

**Distribution**. CAM. Colombia. Costa Rica: Alajuela, Cartago, Guanacaste, Heredia, Limón, Puntarenas, San José. Panama: Bocas del Toro, Chiriquí (Cameron 1893; Dalla Torre 1897; Bradley 1945; Finnamore and Hanson 1995; Ramírez-Guillén et al. 2026).

#### *Lissocampsomeris hoffmanseggii* Klug, 1805

**Distribution**. SAM. Brazil: Distrito Federal. French Guiana. Guyana. Paraguay. (Klug 1805; Spinola 1853; Dalla Torre 1897; Bradley 1945).

**Synonyms**. *Scolia hexaspilota* Spinola, 1853; *Elis ambigua* Sichel, 1864; *Scolia klotzii* Dalla Torre, 1897.

#### *Lissocampsomeris idonea* Bradley, 1945

**Distribution**. SAM. French Guiana: Cayenne (Bradley 1945).

#### *Lissocampsomeris wesmaeli* Lepeletier, 1845

**Distribution**. CAM, NAM, SAM. Bolivia (Est of the Andes). Brazil: Distrito Federal, Mato Grosso do Sul (Chapada). Colombia: Chocó, Meta. Costa Rica: Alajuela, Cartago, Guanacaste, Heredia, Limón, Puntarenas, San José. French Guiana. Guyana. Guatemala: Alta Verapaz. Mexico: Oaxaca, Veracruz. Panama: Chiriquí, Panamá Oeste. Paraguay: Alto Paraná (Puerto Bertoni). Peru: Cusco, Huánuco, Junín, Ucayali. Surinam. Trinidad and Tobago. Venezuela: Apure, Anzoátegui, Distrito Capital, Mérida (Lepeletier 1845; Spinola 1853; Smith 1855; Dalla Torre 1897; Fox 1896; Schrottky 1910; Bradley 1945; Finnamore and Hanson 1995; Fernández and Cubillos. 1999; Dos Santos et al. 2015; Ramírez-Guillén et al. 2026).

**Synonyms**. *Scolia conformis* Spinola, 1853; *Scolia conspicua* Smith, 1855.

### Genus: *Pygodasis* Bradley, 1957

#### *Pygodasis bistrimaculata* Lepeletier, 1845

**Distribution**. SAM. Brazil. Paraguay: Misiones. Uruguay: Montevideo, Rivera (Bradley 1974).

**Synonyms**. *Elis fossor* Saussure, 1858; *Elis talpa* Saussure, 1858.

#### *Pygodasis cineraria* Sichel, 1864

**Distribution**. SAM. Uruguay: Montevideo (Bradley 1974).

#### *Pygodasis colombiensis* Bradley, 1945

**Distribution**. SAM. Colombia: Magdalena, Putumayo, Norte de Santander. Venezuela: Merida (Bradley 1945; Fernández and Cubillos 1999).

#### *Pygodasis cristata* Bradley, 1945

**Distribution**. SAM. Brazil: Bahía. Colombia: Boyacá, Cundinamarca, Santander, Tolima (Ibagué). Ecuador: Morona Santiago (Normandía) (Bradley 1945; Fernández and Cubillos 1999).

#### *Pygodasis ephippium ephippium* Say, 1837

**Distribution**. CAM, NAM, SAM. Colombia: Caldas (Manizales), Cauca*, Tolima, Valle del Cauca. Costa Rica: Alajuela*, Cartago, Guanacaste*, Puntarenas*, San José. Ecuador: Santo Domingo de los Tsáchilas*. Guatemala: Chimaltenango*, Guatemala*, Huehuetenango, Sacatepéquez*. Honduras: El Paraíso*, Francisco Morazán*, Ocotepeque*, Olancho*, Yoro*. French Guiana. Mexico: Chiapas, Coahuila, Estado de Mexico, Guanajuato, Guerrero, Hidalgo, Jalisco, Michoacán, Morelos, Nayarit*, Nuevo León, Oaxaca*, Puebla, Querétaro, Sonora*, Sinaloa, Tamaulipas*, Veracruz, Yucatán. Nicaragua. Panama: Chiriquí* Coclé*, Colón, Panamá*. United States: Louisiana, Texas (Cameron 1893; Bradley 1945; Porter 1981; Finnamore and Hanson 1995; Fernández and Cubillos 1999; Ramírez-Guillén et al. 2022).

**Synonym**. *Scolia petitii* Guérin, 1838.

#### *Pygodasis ephippium wagneriana* Saussure, 1864

**Distribution**. CAM, SAM. Colombia. Costa Rica: Alajuela*, Cartago*, Heredia*, Limón*, Puntarenas*, San José*. Ecuador: Santo Domingo de los Tsáchilas*. Panama: Chiriquí* (Bradley 1945; Añino et al. 2024).

#### *Pygodasis hyalina* Saussure, 1864

**Distribution**. SAM. Argentina: Misiones. Brazil. Colombia. Guyana (Bradley 1945).

**Synonym**. *Elis fallax* Saussure, 1855.

#### *Pygodasis ianthina* Bradley, 1945

**Distribution**. SAM. Brazil: Minas Gerais, Piauí, Río de Janeiro, Rio Grande do Sul, São Paulo. Colombia: Boyacá, Cundinamarca, Magdalena, Meta, Tolima (Guayabal). French Guiana: Cayenne. Venezuela: Aragua, Distrito Capital, Mérida (Bradley 1945; Finnamore and Hanson 1995; Fernández and Cubillos 1999).

#### *Pygodasis lucasia* Saussure, 1858

**Distribution**. SAM. Brazil. Uruguay: Montevideo (Bradley 1974).

#### *Pygodasis quadrimaculata* Fabricius, 1804

**Distribution**. NAM. United States: Florida, Georgia*, Kansas*, Lousiana*, Mississippi, New Jersey*, Oklahoma*, Texas* (Grissell 2007).

**Synonym**. *Colpa pensylvanica* Lepeletier, 1845.

#### *Pygodasis spegazzini* Bréthes, 1910

**Distribution**. SAM. Argentina: Catamarca, Córdoba, Mendoza (Bréthes 1910).

**Synonym**. *Elis bruchii* Bréthes, 1910.

#### *Pygodasis terrestris* Saussure, 1858

**Distribution**. SAM. Argentina: Córdoba. Brazil: Pará (Santarén). Uruguay: Montevideo (Kirby 1889; Fox 1896; Schrottky 1910; Bradley and Betrem 1966; Bradley 1974).

**Synonyms**. *Scolia vidua* Saussure, 1858; *Elis mutanda* Saussure & Sichel, 1858; *Scolia consularis* Burmeister, 1874; *Camposmeris bivittata* Kirby, 1889.

#### *Pygodasis vernoninae* Schrottky, 1910

**Distribution**. SAM. Brazil: Mato Grosso do Sul (Chapada). Paraguay: Alto Paraná (Tacurú Pucú) (Fox 1896; Schrottky 1910).

**Synonym**. *Elis cineraria* Fox, 1896.

#### *Pygodasis vespiformis* Saussure, 1858

**Distribution**. SAM. Brazil: Distrito Federal, Minas Gerais (Las Minas). Uruguay (Bradley 1974).

#### *Pygodasis vittata vittata* Sichel, 1864

**Distribution**. CAM, SAM. Argentina: Entre Ríos (Paraná), Tucumán. Brazil. Panama. Paraguay: Alto Paraná (Tacurú Pucú) (Bréthes 1910; Schrottky 1910; Bradley 1945, 1974; Finnamore and Hanson 1995).

**Synonym**. *Scolia argentina* Bréthes, 1910.

#### *Pygodasis vittata banksi* Bradley, 1945

**Distribution**. CAM, SAM. Colombia: Magdalena. Panama: Panama (Vella vista) (Bradley 1945).

### Genus: *Rhabdotomeris* Bradley, 1957

#### *Rhabdotomeris rokitanskyi* Dalla Torre, 1897

**Distribution**. CAM, NAM, SAM. Costa Rica: Guanacaste*, Heredia*, San José*. Guatemala*: Chimaltenango, Guatemala. Mexico: Chiapas, Estado de Mexico*, Guanajuato, Guerrero, Jalisco*, Michoacán, Morelos, Nuevo León*, Oaxaca*, Puebla*. From Mexico to Ecuador (Cameron 1893; Bradley 1957; Finnamore and Hanson 1995; Ramírez-Guillén et al. 2022).

**Synonym**. *Elis mexicana* Cameron, 1893.

### Genus: *Sphenocampsomeris* Bradley, 1957

#### *Sphenocampsomeris obesa* Saussure, 1869

**Distribution**. SAM. Argentina. Brazil. Uruguay (Montevideo) (Bradley 1957, 1974).

### Genus: *Stygocampsomeris* Bradley, 1957

#### *Stygocampsomeris corrigenda* Schulz, 1906

**Distribution**. SAM. Brazil: Mato Grosso do Sul (Chapada), Pará. Paraguay (Fox 1896; Schrottky 1910; Bradley 1974).

**Synonym**. *Elis nigra* Saussure, 1858.

#### *Stygocampsomeris sanktae-theresae* Bradley, 1945

**Distribution**. SAM. Brazil: Espirito Santo, Minas Gerais, Rio de Janeiro. Colombia: Valle del Cauca. French Guiana: Saint-Laurent-du-Maroni (Bradley 1945; Fernández and Cubillos 1999).

#### *Stygocampsomeris servillei* Guérin, 1838

**Distribution**. ANT, CAM, NAM, SAM. Argentina: Buenos Aires, Misiones. Bolivia (Oeste a la costa). Brazil: Mato Grosso do Sul (Chapada). Chile: Arica. Colombia: Amazonas, Antioquia, Atlántico, Boyacá, Caquetá, Casanare, Cauca, Chocó, Cundinamarca, Magdalena, Meta, Santander, Valle del Cauca, Vichada. Costa Rica: Alajuela*, Cartago*, Guanacaste*, Heredia*, Limón* Puntarenas*, San José*. Guatemala: Alta Verapaz*, Baja Verapaz*, Huehuetenango, Izabal*. French Guiana. Mexico: Chiapas, Veracruz*. Panama: Chiriquí* Coclé*, Colón*, Los Santos*, Panamá*, Panamá Oeste*. Paraguay. Peru: Lima. Suriname. Trinidad and Tobago. Uruguay. Venezuela: Aragua, Mérida, Monagas (Fox 1896; Schrottky 1910; Bradley 1940, 1945; Finnamore and Hanson 1995; Fernández and Cubillos 1999; Rasmussen and Asenjo 2009; Ramírez-Guillén et al. 2022).

**Note**. In Bradley (1973), under the last reference, he mentions that *S. servillei* should be dated 1838 and not 1831. We only found the reference from Guérin (1830).

**Synonyms**. *Campsomeris hyalina* Lepeletier, 1845; *Dielis neotropica* Gribodo, 1895.

#### *Stygocampsomeris manauara* Golfetti, 2023

**Distribution**. SAM. Brazil: Amazonas (Golfetti and Noll 2023b).

### Genus: *Tenebromeris* Betrem, 1963

#### *Tenebromeris tenebrica* Bradley, 1957

**Distribution**. SAM. Brazil: Minas Gerais (Betrem 1963; Bradley 1957).

### Genus: *Xanthocampsomeris* Bradley, 1957

#### *Xanthocampsomeris completa* Rohwer, 1927

**Distribution**. CAM, NAM. Costa Rica*: Alajuela, Guanacaste, San José. Guatemala: Guatemala*, Izabal. El Salvador: San Salvador*. Honduras: Comayagua, El Paraíso*, Francisco Morazán*. Mexico: Chiapas, Estado de Mexico, Guanajuato, Guerrero, Hidalgo, Jalisco, Michoacán, Morelos, Nuevo León*, Oaxaca, Puebla, Querétaro, San Luis Potosí, Sinaloa, Sonora*, Tamaulipas (Victoria). United States: Arizona, New Mexico*, Texas, Utah* (Rowher 1927; Mackay 1987; Ramírez-Guillén et al. 2022).

**New synonym**. *Xanthocampsomeris completa yucatanensis* Bradley, 1964.

**Note**. We are synonymizing *Xanthocampsomeris completa yucatanensis* Bradley, 1964 as *Xanthocampsomeris completa*.

#### *Xanthocampsomeris fulvohirta* Cresson, 1865

**Distribution**. ANT, NAM. Cuba: Artemisa (Taco Taco) Santiago de Cuba. Jamaica: Portland. United States: Florida (Rowher 1927; Portuondo-Ferrer and Fernández-Triana 2004).

#### *Xanthocampsomeris hesterae* Rohwer, 1927

**Distribution**. CAM, NAM, SAM. Colombia: Antioquia, Cesar, Cundinamarca, Santander, Tolima, Valle del Cauca. Costa Rica: Alajuela*, Cartago*, Guanacaste*, Heredia*, Limón, Puntarenas, San José*. Ecuador. Guatemala: Alta Verapaz*, Huehuetenango, Izabal (Cayuga). Honduras*: Atlántida, Francisco Morazán, Olancho, Yoro. Mexico: Campeche* Chiapas, Oaxaca*, Quintana Roo, Tabasco, Veracruz. Panama: Chiriquí* Coclé*, Colón*, Darién*, Los Santos*, Panamá*, Panamá Oeste*. Trinidad y Tobago: Tunapuna-Piarco (Tucuche). United States: Texas. Venezuela: Distrito Capital (Adjuntas, Caracas), Mérida, La Guaira (Galipán) (Rowher 1927; Bradley 1945; Porter 1981; Finnamore and Hanson 1995; Fernández and Cubillos 1999; Ramírez-Guillén et al. 2022).

#### *Xanthocampsomeris limosa* Burmeister, 1854

**Distribution**. CAM, NAM. Guatemala*: Sacatepéquez, Sololá. Mexico: Aguascalientes, Campeche, Chiapas, Ciudad de Mexico*, Chihuahua, Ciudad de Mexico, Coahuila, Durango, Estado de Mexico, Guanajuato, Guerrero* Hidalgo*, Jalisco, Michoacán, Morelos, Nayarit, Nuevo León*, Oaxaca, Puebla, Querétaro, Tamaulipas, Tlaxcala, San Luis Potosí, Veracruz, Zacatecas*. United States: Arizona (Rowher 1927; Mackay 1987; Ramírez-Guillén and Falcon-Brindis 2022; Ramírez-Guillén et al. 2022).

#### *Xanthocampsomeris tricincta* Fabricius, 1775

**Distribution**. ANT. Cuba. Haiti: Oeste (Port-au-Prince). Puerto Rico: Aguadilla/Mayagüez, Aguas Buenas*, Arecibo (Manatí), Carolina (Luquillo), Humacao (Naguabo), Guayama (Aibonito, Cayey), Isabela*, Lajas*, San Juan (San Juan, Santa Rita), Ponce (Adjuntas Mameyes, Maricao) (Gundlach 1887; Rowher 1927; Wolcott 1948).

**Synonyms**. *Scolia undulata* Smith, 1855; *Campsomeris pyrura* Rohwer, 1927.

### Subtribe: Trielidina Betrem, 1972

**Genus: *Colpa* Dufour, 1841**

#### *Colpa octomaculata octomaculata* Say, 1823

**Distribution**. NAM. United States: Colorado, Illinois, Kansas, Minnesota, Nebraska, North Dakota, Oklahoma, South Dakota, Texas, Wyoming (Bradley 1928a).

#### *Colpa octomaculata hermione* Banks, 1912

**Distribution**. NAM. United States: Alabama, Florida, Georgia, Illinois, North Carolina (Bradley 1928a; Grissell 2007).

#### *Colpa octomaculata texensis* Saussure, 1858

**Distribution**. NAM. Mexico: Baja California, Baja California Sur, Chihuahua, San Luis Potosí, Sonora. United States: Arizona, California, Colorado, Kansas, New Mexico, Texas (Bradley 1928a; Hurd 1952; Porter 1981; Ramírez-Guillén et al. 2022).

**Synonyms**. *Scolia consors* Cresson, 1865; *Scolia flavosignata* Cresson, 1865; *Scolia lupina* Cresson, 1865; *Scolia regina* Cresson, 1865; *Elis zonzaria* Cresson, 1865.

#### *Colpa octomaculata xantiana* Saussure, 1864

**Distribution**. NAM. Mexico: Baja California, Baja California Sur (Cabo San Lucas) (Bradley 1928a, 1974).

#### *Colpa pollenifera* Viereck, 1906

**Distribution**. NAM. Mexico: Sonora. United States: Arizona, Colorado, Kansas, New Mexico, Texas* (Bradley 1928a; Porter 1981).

### Genus: *Crioscolia* Bradley, 1951

#### *Crioscolia alcione* Banks, 1917

**Distribution**. NAM. Mexico: Baja California, Nuevo León*, Sonora*, Tamaulipas*. United States: Arizona*, California, Colorado, Idaho*, Nevada, New Mexico, Oregon*, Utah, Washington (Bradley 1928a; Hurd 1952; MacKay 1987; Ramírez-Guillén et al. 2022).

#### *Crioscolia flammicoma* Bradley, 1928

**Distribution**. NAM. Mexico: Baja California, Sonora. United States: Arizona, California, Nevada*, New Mexico (Bradley 1928a; Hurd 1952; MacKay 1987; Ramírez-Guillén et al. 2022).

### Tribe: Scolinii Latreille, 1802

**Genus: *Scolia* Fabricius, 1775**

#### *Scolia albumtenebris* Ramírez-Guillén, 2025

**Distribution**. CAM. Panama: Darién, Panamá, Panamá Oeste (Ramírez-Guillén et al. 2025c).

#### *Scolia bartletti* Ramírez-Guillén, 2025

**Distribution**. NAM. Mexico: Chiapas, Guanajuato, Guerrero, Michoacán, Oaxaca (Ramírez-Guillén et al. 2025c).

#### *Scolia bicincta* Fabricius, 1775

**Distribution**. NAM. United States: Florida, Massachusetts, Mississippi*, Missouri*, North Carolina*, Texas, Virginia* (Bartlett 1912; Grissell 2007).

**Synonyms**. *Scolia obscura* Klug, 1805; *Scolia undata* Klug, 1810; *Scolia bifasciata* Smith, 1855.

**Note**. This taxon probably refers to two sibling species, one not yet described mentioned in the work in press by Khouri et al. 2022.

#### *Scolia binominata* Schulz, 1906

**Distribution**. SAM. Brazil: Mato Grosso (Schulz 1906).

#### *Scolia cubensis* Bartlett, 1912

**Distribution**. ANT. Cuba (Bartlett 1912).

#### *Scolia dubia dubia* Say, 1837

**Distribution**. NAM. Mexico. United States: Arizona, Carolina, Delaware*, District of Columbia*, Florida, Georgia, Louisiana, Massachusetts, Maryland, Missouri*, New York, Noth Carolina*, Pennsylvania*, Tennessee, Texas, Virginia (Bartlett 1912; MacKay 1987; Grissell 2007).

**Synonyms**. *Scolia aulica* Burmeister, 1854; *Scolia thalia* Banks, 1912.

#### *Scolia dubia haematodes* Burmeister, 1854

**Distribution**. NAM. Mexico: Coahuila. United States: Arizona, California, Nuevo Mexico, Texas (Bartlett 1912; Hurd 1952; MacKay 1987; Ramírez-Guillén et al. 2022).

**Synonym**. *Scolia americana* Saussure, 1857.

#### *Scolia fuscipennis* Bartlett, 1912

**Distribution**. NAM. Mexico: Veracruz (Bartlett 1912; Ramírez-Guillén et al. 2022).

#### *Scolia guttata* Burmeister, 1854

**Distribution**. ANT, CAM, NAM. Colombia. Costa Rica: Cartago. Guatemala: Baja Verapaz. Mexico: Chiapas, Ciudad de Mexico*, Coahuila, Colima*, Durango*, Guanajuato, Guerrero* Hidalgo, Jalisco, Michoacán, Morelos* Nayarit, Nuevo León, Oaxaca, Puebla, Querétaro, San Luis Potosí, Tamaulipas: (Tampico), Tlaxcala*, Veracruz. Nicaragua: Chontales. Panama: Chiriquí. Trinidad and Tobago. United States: Arizona*, New Mexico, Texas. Venezuela: Distrito Capital (Caracas) (Kirby 1889; Cameron 1893; Bartlett 1912; MacKay 1987; Ramírez-Guillén et al. 2022).

**Synonyms**. *Discolia hecate* Kirby, 1889; *Scolia tristis* Burmeister, 1854; Scolia guttata var. eximia Smith, 1855, syn. nov.; *Scolia azteca* Saussure, 1857.

**Note**. ICZN does not recognize variety names. Scolia guttata var. eximia should no longer be used.

#### *Scolia jucunda* Saussure, 1858

**Distribution**. SAM. Brazil: Brasilia. Uruguay: Montevideo (Bradley 1974).

#### *Scolia mexicana* Saussure, 1858

**Distribution**. NAM. Mexico: Estado de Mexico, Chiapas, Coahuila, Guanajuato, Guerrero, Michoacán, Morelos, Oaxaca*, Querétaro. United States: Texas, Arizona (Cameron 1893; MacKay 1987; Ramírez-Guillén et al. 2022).

**Synonyms**. *Scolia monticola* Cameron, 1873; *Scolia nigrescens* Bartlett, 1912.

#### *Scolia nobilitata nobilitata* Fabricius, 1804

**Distribution**. NAM. United States: Alabama*, Arizona, Carolina, Florida, Georgia, Illinois*, Long Island, Mississippi* Missouri*, New York, North Carolina, Pennsylvania, Tennessee*, Texas, Virginia (Bartlett 1912).

**Synonyms**. *Sphex bifasciata* Swederus nomen dubium?; *Scolia tricolor* Klug, 1805; *Scolia maculata* Guérin, 1838; *Scolia ornata* Smith, 1855.

#### *Scolia nobilitata otomita* Saussure, 1858

**Distribution**. NAM. United States: Alabama*, Arizona, California*, Florida, New Mexico*, Texas*. Mexico: Baja California Sur* (Bartlett 1912; Bradley 1974).

**Synonym**. *Scolia fulviventris* Bartlett, 1912.

#### Scolia nobilitata otomita var. consors Saussure, 1863

**Distribution**. NAM. Mexico: Baja California Sur (Cabo San Lucas) (Bradley 1974).

**Synonym**. *Scolia amoena* Cresson, 1856.

#### *Scolia nobilitata tricincta* Say, 1823

**Distribution**. NAM. United States: Arizona*, California, Colorado, Kansas, New Mexico, Oklahoma*, Texas. Mexico*: Baja California Sur, Chihuahua, Coahuila, Durango, Nuevo León, San Luis Potosí (Bartlett 1912).

**Synonyms**. *Scolia ridingsii* Cresson, 1865; *Scolia inconstans* Cresson, 1865; *Scolia flavocostalis* Cresson, 1868; *Scolia lecontei* Cresson, 1868; *Scolia lewisii* Cresson, 1868.

#### *Scolia rufiventris* Fabricius, 1804

**Distribution**. CAM, NAM, SAM. Brazil: Mato Grosso, Minas Gerais. Colombia. Costa Rica: Guanacaste, Limón, Puntarenas. Guyana. Honduras: Comayagua. Mexico. Panama: Panama. Peru: Junín, Ucayalí (Fabricius 1804; Fox 1896; Bradley 1974; Dos Santos et al. 2015; Añino et al. 2020; Aranda 2021; Ramírez-Guillén et al. 2022).

**Synonyms**. *Scolia anceps* Saussure, 1858; *Scolia drewseni*, 1858.

#### *Scolia scutata* Bréthes, 1910

**Distribution**. SAM. Argentina: Misiones (Iguazú) (Bréthes 1910).

#### *Scolia vintschgaui* Dalla Torre, 1893

**Distribution**. NAM. Mexico: Baja California Sur, Guanajuato, Guerrero, Jalisco, Oaxaca*, San Luis Potosí*, Sonora*. United States*: Arizona, New Mexico (Cameron 1893; Bartlett 1912; Ramírez-Guillén et al. 2022).

**Synonym**. *Scolia saussurei* Cameron, 1893.

### Genus: *Triscolia* Saussure, 1863

#### *Triscolia ardens* Smith, 1855

**Distribution**. NAM. Mexico: Querétaro, San Luis Potosí, Sonora. United States: Arizona, California, New Mexico, Texas, Utah* (Cameron 1893; Bartlett 1912; Hurd 1952; MacKay 1987; Ramírez-Guillén et al. 2022).

**Synonyms**. *Scolia fervida* Burmeister, 1854; *Scolia montezumae* Saussure, 1857.

#### *Triscolia badia* Saussure, 1863

**Distribution**. NAM. Mexico: Baja California, Baja California Sur (Cabo San Lucas). United States (Bartlett 1912; Betrem and Bradley 1964; Bradley 1974; Ramírez-Guillén et al. 2022).

### Species with dubious names

#### *Colpa? lugens* Kirby, 1889

**Distribution**. SAM. Brazil: Rio Grande do Sul (Kirby 1889; Bradley 1928a).

#### *Micromeriella*? *nana* Saussure, 1867

**Distribution**. SAM Brazil (Bradley 1974).

#### *Campsomeris*? *nigrans* Bradley, 1945

**Distribution**. SAM. Argentina: Misiones, Santa Fe. Bolivia: Santa Cruz. Brazil: Goiás, Matto Grosso, Rio de Janeiro, Rio Grande do Sul, São Paulo. Colombia: Cesar (Chiriguaná). French Guiana: Cayenne (Bradley 1945).

**Synonyms**. Female of *hyalina* Saussure, 1864? (*Elis*); *Campsomeris nigra* Saussure, 1858?; *Scolia corrigenda* Schulz, 1906?

#### *Scolia decepta* Fox, 1896

**Distribution**. SAM. Brazil: Mato Grosso do Sul (Chapada) (Fox 1896).

#### *Scolia nigrescens* Fox, 1896

**Distribution**. SAM. Brazil (Fox 1896).

## Discussion

This work provides the first species checklist of the Scoliidae occurring in the Americas. After significant field sampling, museum specimen documentation and literature search in this study and previous work by Ramírez-Guillén et al. 2022 (38 collections visited and 8,072 examined specimens), we report 72 species and provide information on their distribution. Currently, taxonomic keys at the regional level are available for Costa Rica, Mexico, the United States, and Venezuela (Bradley 1945; Hurd 1952; Porter 1981; MacKay 1987; Finnamore and Hanson 1995; Grissell 2007; Ramírez-Guillén et al. 2022), though some are outdated. Other works including taxonomic keys have been reported for *Aelocampsomeris*, *Colpa*, *Crioscolia*, *Dielis*, *Lissocampsomeris*, *Scolia*, *Triscolia* and *Xanthocampsomeris* (Bartlett 1912; Rowher 1927; Bradley 1928a, 1928b, 1957; Castagnet et al. 2025; Ramírez-Guillén et al. 2025c, 2026).

Brazil (*n* = 32), Mexico (*n* = 29), and the United States (*n* = 24) are the top three countries with the highest number of species recorded. However, the research efforts in these regions have been variable. The United States holds by far the highest number of published taxonomic works (Bartlett 1912; Rowher 1927; Bradley 1928a, 1928b; Betrem and Bradley 1964; Porter 1981; MacKay 1987; Grissell 2007; Szafranski 2023, 2026). In Mexico, recent works on Scoliidae have provided insights into this fauna, including descriptions of new species of *Scolia* (Ramírez-Guillén and Falcon-Brindis 2022; Ramírez-Guillén et al. 2022, 2025b, 2025c). Likewise, the Brazilian scoliids are receiving more attention, and new species of *Dielis* and *Stygocampsomeris* have been recently described (Golfetti and Noll 2023a, 2023b).

Despite these recent efforts, Scoliidae in South America remain understudied. The fauna of Brazil and neighboring countries, such as Argentina, Bolivia, Chile, Paraguay, and Uruguay, remains poorly understood. Most taxonomic revisions are more than 100 years old and focus primarily on Argentina and Brazil (Fox 1896; Bréthes 1910). Notably, there is a lack of records in Belize and only very few records from El Salvador (*n* = 3) and Nicaragua (*n* = 4), which limits our understanding of scoliids in Central America. In contrast, countries such as Costa Rica (*n* = 14), Guatemala (*n* = 12), Honduras (*n* = 7), and Panama (*n* = 14) show higher numbers of records, likely reflecting increased recent interest in these wasps (Añino et al. 2020, 2024; Ramírez-Guillén et al. 2025a, 2025c, 2026).

### Status of subspecies

Of the 72 listed species, 11 have at least one subspecies. Of the 11 species, six are distributed in North America, and their respective subspecies are all found in the United States. Historically, subspecies have been proposed based on the number of spots on the terga, setae coloration, or wing color reflection. Only the subspecies in *Dielis plumipes* show morphological differences. This situation is probably what originated a convoluted classification for the group, where previous entomologists proposed new subspecies based on coloration patterns, which is not necessarily reliable for a group like Scoliidae, which exhibit various pigmentation patterns and a wide range in body size. The biggest limitation for phylogenetic approaches to resolving these ambiguities is the scarcity of freshly collected specimens and museum specimens preserved in optimal conditions. In addition, collecting scoliid wasps in the field can be challenging, as their plant foraging preferences are unknown, and most of their insect hosts remain a mystery (Ramírez-Guillén et al. 2025b).

In some cases, the distribution might be helpful to separate species with subjective pigmentation patterns. For example, *Pygodasis ephippium ephippium* and *P. ephippium wagneriana* are separated by wing color variation: metallic violaceous reflections and orange hyaline, respectively. *P. e. wagneriana* is found in southern Costa Rica, Panama, Colombia, and Ecuador, while *P. e. ephippium* is found from the southern United States to Argentina (Cameron 1893; Bradley 1945; Porter 1981; Finnamore and Hanson 1995; Fernández and Cubillos 1999; Ramírez-Guillén et al. 2022; Añino et al. 2024).

Another example is *Lissocampsomeris columba columba* and *L. columba albofimbriata*, which show color variation in the setae and wings. *Lissocampsomeris columba columba* has been recorded in Mexico, Guatemala, Colombia, and Venezuela, while *L. columba albofimbriata* occurs in Costa Rica, Panama, and Colombia (Ramírez-Guillén et al. 2026).

Another issue that has not been discussed before is the designation of varieties, especially among North American species. In general, taxa with variation (var.) status should no longer be used, as it is highly questionable given the lack of robust morphological support. For example, Scolia guttata var. eximia (proposed by Bartlett 1912) are most likely variations of *Scolia guttata*, thus we suggest only using the latter in future works. This species exhibits high color variation across the tergal segments, ranging from individuals with yellow spots on T2–T3, T2–T4, T1–T4, T1–T5 to immaculate.

We suggest *Scolia nobilitata otomita* and *Scolia nobilitata tricincta* as synonyms of *Scolia nobilitata*, as they have overlapping distributions and have been separated based on the number of yellow tergal spots. Likewise, we suspect that Scolia nobilitata otomita var. consors corresponds to *Scolia vintschgaui*, as these species have overlapping distributions similar tergal markings. However, we decided to keep both of them in this list since we did not revise enough specimens to make such claim.

We suggest *Colpa octomaculata texensis* and *Colpa octomaculata hermione* as synonyms of *Colpa octomaculata*. The distinction between these taxa is based on the red or black coloration present on the body and legs of males and females. Even in taxonomic keys, there is no distinction between males of *C. o. octomaculata* and *Colpa o. texensis*. Furthermore, *Colpa octomaculata xantiana* is morphologically distinct; it is mentioned that it has a scutellum divided by a median sulcus, a characteristic shared with *Colpa pollenifera*. This raises questions whether *C. o. xantiana* is a synonym of *Colpa octomaculata*, a separate species, a variation of *C. pollenifera*, or a taxonomic mistake. Moreover, the male of *C. o. xantiana* has not been described, further increasing the uncertainty about its classification.

*Scolia dubia dubia* and *Scolia dubia haematodes*, as they have a clearly allopatric distribution. *Scolia dubia dubia* is distributed in central and eastern United States, whereas *S. dubia haematodes* is distributed from central Mexico (Querétaro) to southwestern United States (Arizona, Nuevo Mexico, and Texas) (Bartlett 1912; Hurd 1952; MacKay 1987; Grissell 2007; Ramírez-Guillén et al. 2022). Given the lack of robust evidence to separate these species, we suggest synonymizing them as *Scolia dubia*. However, since *S. dubia dubia* and *S. dubia haematodes* have a distinct distribution, a thorough molecular or morphological analysis is necessary.

We did not examine enough specimens of *Dielis trifasciata trifasciata* and *D. trifasciata nassauensis* to forge an opinion on their status. We only checked one male and one female from each taxon. The only difference to separate these subspecies has traditionally been the width of the bands on T1–T3. We could not review any specimens of *Dielis bahamensis*. It has been suggested that *D. bahamensis*, *D. trifasciata nassauensis*, and *D. trifasciata trifasciata* represent a single diverse population within one taxon (Beaty et al. 2009).

*Dielis plumipes* might be a species complex, as the subspecies show distinct morphological differences, but the distribution patterns are unclear. We maintain the subspecies status of *Dielis plumipes plumipes*, *Dielis plumipes confluenta*, and *Dielis plumipes fossulana* although it is necessary to conduct an integrative approach to study these subspecies.

*Xanthocampsomeris completa yucatanensis* is established as a synonym of *Xanthocampsomeris completa*. Bradley (1964a) indicated that *X. c. yucatanensis* is a dubious name for the taxon designated by Rohwer. The description of this species is attributed to Bradley, who mentioned that the only difference between *X. c. yucatanensis* and *X. c. completa* is the interruption of the tergite bands. Only females have been recorded in Yucatan, Mexico. We did not examine females of this subspecies and males have not been described.

We have not seen specimens of *Lissocampsomeris aureohirta aureohirta*, *L. aureohirta ecuadorensis*, *Pygodasis vittata vittata*, and *P. vittata banksi*. Like the other subspecies, they need to be revised.

### Erroneous records

*Megacampsomeris drewseni* was listed by Osten (2005) to occur in the Neotropical region, but Bradley (1974) mentioned that this species occurs in the Indo-Malayan region. Therefore, this species was excluded from the above list. In Osten’s (2005) checklist, it is indicated that *Campsomeris whitelyi* and *Lissocampsomeris chilensis* are placed in incorrect genera, and they should be listed as *Dielis chilensis* and *Dielis whitelyi*, previously listed by Bradley (1957). Finnamore and Hanson (1995) mentioned that *Dielis plumipes* is found in Costa Rica, but this species is restricted to North America. The genus *Phalerimeris* was listed for Mexico in Ruíz and Coronado (2002). However, it is not present in the Americas and is found, instead, in the Indo-Australian region. *Stygocampsomeris servillei* is present in Chile according to Pizarro-Araya et al. (2021), but this is incorrect and corresponds to a *Dielis* sp. *Scolia albumtenebris* was identified as *Scolia guttata* in Añino et al. (2020), but it is more likely that the specimens mentioned in this work for Costa Rica and Panama belong to *S. albumtenebris* (Ramírez-Guillén et al. 2025c).

Ruíz et al. (2010) listed *Dielis plumipes* for the state of Tamaulipas. Due to its distribution mentioned in Bradley (1928b) and (Szafranski 2026), it is likely to be the subspecies *D. plumipes fossulana*, *D. tejensis* or *D. tolteca*. We placed this record in *D. plumipes fossulana* with a question mark. However, we have not reviewed any specimens of *D. plumipes* in the collections that come from Mexico. There is no record of *Scolia bicincta* in Mexico, as noted in Ramírez-Guillén et al. (2022). This publication also listed *Pygodasis hyalina* as occurring in Mexico and Añino et al. (2024) listed this species from Panama. Both records are erroneous and correspond to *Lissocampsomeris columba columba* and *L. columba albofimbriata*, respectively (Ramírez-Guillén et al. 2026). *Pygodasis ianthina* is distributed in South America, but Bradley (1945) suggested that the single known specimen from Mexico should be viewed with caution and must be verified with additional material from records of the country. Bradley (1945) also mentioned *Pygodasis vittata vittata* and *P. vittata banksi* as occurring in Mexico and Panama, respectively. However, neither of these subspecies has been found in Mexico or Central America. We rectify the distribution of *Xanthocampsomeris tricincta*, which is distributed in Cuba, Haiti, and Puerto Rico, but not in Mexico. Rowher (1927) had already suggested that it might be a misidentification.

### Missing opposite genders

*Scolia fuscipennis* was described in 1912, and the female remains unknown. It should be noted that we have only seen a few males, all coming from the type locality in Cordoba, Veracruz, Mexico (Bartlett 1912; Ramírez-Guillén et al. 2022). The male of *Scolia bartletti* and female of *Lissocampsomeris bribri* were described in 2025 and 2026, but their female and male, respectively, remain unknown (Ramírez-Guillén et al. 2025c, 2026).

### Gynandromorph specimens

Four gynandromorph specimens are known in Scoliidae. *Colpa sexmaculata* (Fabricius, 1781) shows mosaic gynandromorphism, and was collected in the Old World at an unknown location (Romand, 1835). *Pygodasis ephippium ephippium* exhibits bilateral gynandromorphism and was collected in Michoacan, Mexico (Krombein 1949). *Xanthocampsomeris limosa* with mosaic gynandromorphism, collected in Tlaxcala, Mexico (Ramírez-Guillén and Falcon-Brindis 2022). *Dielis tolteca* with a mosaic gynandromorphism, was collected in Costa Rica (Ramírez-Guillén et al. 2025a).

### Endemism

A total of 19 country endemic species occur in the Americas (Table 2), Brazil holding the largest number of species. Interestingly, many of these records are located near the border with neighboring countries. This is also the case of Argentina, French Guiana, Peru, and Uruguay. *Lissocampsomeris bribri* has been recorded only in Panama from a single female specimen (Ramírez-Guillén et al. 2026). However, we acknowledge that the endemism found in this work may be an artifact of collecting efforts.

## Conclusions

This work updates and establishes a list of current valid species. Monographic approaches will facilitate information and help to identify the gaps that need to be addressed in this group of wasps. The status of the scoliid fauna in the Americas is strongly influenced by region-specific taxonomic efforts, which have advanced our knowledge in the United States, Mexico, and Brazil. Although this pattern is not unique to Scoliidae, it represents an important gap for conservation efforts (Falcon-Brindis et al. 2021). More sampling efforts are necessary in underrepresented countries such as: Argentina, Bolivia, Chile, El Salvador, Nicaragua, Paraguay, Uruguay and especially in Belize. This is necessary to generate a user-friendly taxonomic key to the American species; even though the subspecies status may later be revised, and provide a photographic catalog showing the main differences between the species. We strongly encourage future works to adopt integrative approaches and include both morphological and molecular data.

## Additional information

### Artificial Intelligence (AI) use

The authors accept full responsibility for the content of the manuscript, including the disclosure of any use of AI.

No AI tools were used in the preparation of this manuscript.

### Author ORCIDs

L.D. Ramírez-Guillén https://orcid.org/0000-0003-2853-2714

J.L. León-Cortés https://orcid.org/0000-0003-1706-5620

L. Leblanc https://orcid.org/0000-0002-6060-1364

A. Falcon-Brindis https://orcid.org/0000-0003-2496-2178

### Data availability

All of the data that support the findings of this study are available in the main text.
